# Molecular and Morphological Evidence Reveals a New Species in the *Phyllomedusa hypochondrialis* Group (Hylidae, Phyllomedusinae) from the Atlantic Forest of the Highlands of Southern Brazil

**DOI:** 10.1371/journal.pone.0105608

**Published:** 2014-08-20

**Authors:** Daniel P. Bruschi, Elaine M. Lucas, Paulo C. A. Garcia, Shirlei M. Recco-Pimentel

**Affiliations:** 1 Departamento de Biologia Estrutural e Funcional, Instituto de Biologia, Universidade Estadual de Campinas - UNICAMP, Campinas, São Paulo, Brazil; 2 Área de Ciências Exatas e Ambientais/Mestrado em Ciências Ambientais, Universidade Comunitária da Região de Chapecó - UNOCHAPECÓ, Chapecó, Santa Catarina, Brazil; 3 Departamento de Zoologia, Instituto de Ciências Biológicas, Universidade Federal de Minas Gerais - UFMG, Belo Horizonte, Minas Gerais, Brazil; Leibniz-Institute of Freshwater Ecology and Inland Fisheries, Germany

## Abstract

The taxonomic status of a disjunctive population of *Phyllomedusa* from southern Brazil was diagnosed using molecular, chromosomal, and morphological approaches, which resulted in the recognition of a new species of the *P. hypochondrialis* group. Here, we describe *P. rustica* sp. n. from the Atlantic Forest biome, found in natural highland grassland formations on a plateau in the south of Brazil. Phylogenetic inferences placed *P. rustica* sp. n. in a subclade that includes *P. rhodei* + all the highland species of the clade. Chromosomal morphology is conservative, supporting the inference of homologies among the karyotypes of the species of this genus. *Phyllomedusa rustica* is apparently restricted to its type-locality, and we discuss the potential impact on the strategies applied to the conservation of the natural grassland formations found within the Brazilian Atlantic Forest biome in southern Brazil. We suggest that conservation strategies should be modified to guarantee the preservation of this species.

## Introduction

The genus *Phyllomedusa* Wagler, 1930 (Anura, Hylidae, Phyllomedusinae) is endemic to the Neotropical region and is currently composed of 30 recognized species [1]. Panama represents the northernmost extreme of the geographic range of this genus, while Argentina and Uruguay constitute its southern limit; species is also being found throughout Colombia, east of the Andes, and in Trinidad [1].

The most recent hypothesis on the phylogenetic relationships of the Phyllomedusinae was presented by Faivovich et al. [2], who recognized the four phenetic groups assigned in the genus as monophyletic groups: the *P. hypochondrialis* group [3], *P. tarsius* group [4], *P. burmeisteri* group [5] and *P. perinesos* group [6]. Nevertheless, some members of this genus have yet to be assigned to a species group [1], [2].

A complex taxonomic scenario has been noted in most species of the *P. hypochondrialis* group [2], [7], [8]. Molecular inferences have revealed two well-supported clades within this group. One subclade includes *P. palliata*, *P. azurea*, *P. hypochondrialis* and *P. nordestina,* while the second comprise *P. rhodei*, *P. ayeaye*, *P. centralis*, *P. megacephala* and *P. oreades* [2].

The use of both morphological and molecular methods is an established and effective approach for the identification of cryptic biodiversity and the clarification of taxonomic uncertainties (e.g. see references [9]–[10]). Speciation may not always be accompanied by morphological changes [11]–[12], in which case the recognition of species might be hampered by the absence of discrete phenotypic traits. The recognition of hidden biodiversity is fundamental for conservation efforts [13], especially the identification of new taxonomic units with small geographic ranges that potentially represent evolutionarily vulnerable lineages [13].

Lucas et al. [14] recorded the presence of a population of *Phyllomedusa* morphologically similar to *P. azurea* in natural grassland formations on a plateau in the highlands of southern Brazil (municipality of Água Doce in Santa Catarina state), but its taxonomic status was unclear. Despite the similar morphology, the authors detected some differences in coloration in comparison with the formal diagnosis of *P. azurea* [3]. Furthermore, this population was found in an unusual location distinct from the savanna formations in which *P. azurea* is known to occur. Bruschi et al. [8] evaluated the taxonomic status of the populations assigned to *P. hypochondrialis* and *P. azurea* from a number of Brazilian regions and included tissue of two specimens from Água Doce. Interestingly, these authors found that the Água Doce population was paraphyletic in relation to the other haplotypes of the *P. azurea* clade, indicating that a more robust analysis, based on a larger number of characters (morphological and genetic) would be needed to identify the true taxonomic status of this population.

In this study, we used morphological, chromosomal and molecular phylogenetic approaches to investigate the taxonomic status of a distinct population of *Phyllomedusa* from southern Brazil. We describe a new species of the genus and infer its phylogenetic relationship as well as present chromosomal data.

## Materials and Methods

### Population sampling

**Figure 1:**
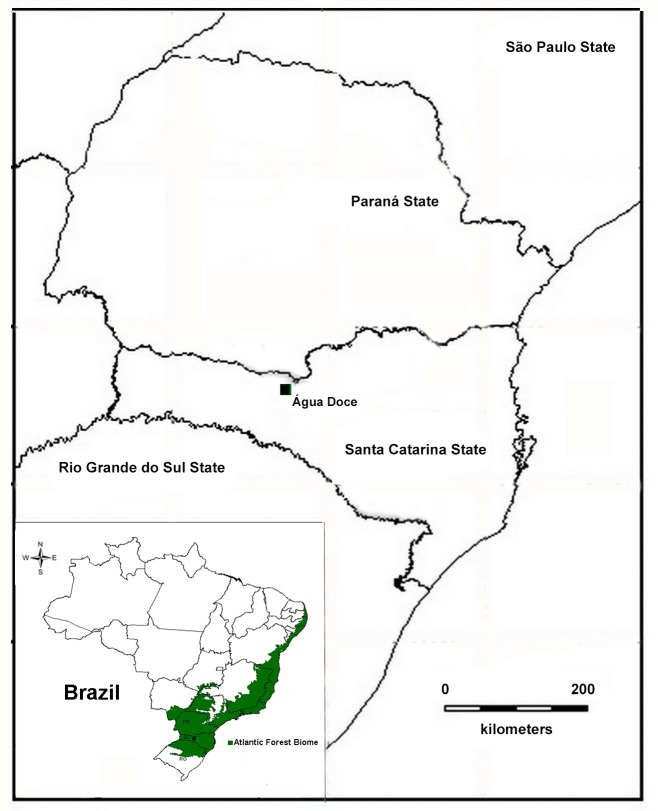
Map of Brazil showing the Atlantic Forest domain (green area). Note the type locality of *Phyllomedusa rustica* sp. n. (square symbol) in the Água Doce municipality, Santa Catarina state.

The individuals examined were collected under authorization number 14468-1/14468-4 issued by SISBIO/Instituto Chico Mendes de Conservação da Biodiversidade. All tissue samples were extracted from euthanized specimens using anesthetic application to the skin (5% Lidocaine) to minimize animal suffering, according to recommendations of the Herpetological Animal Care and Use Committee (HACC) of the American Society of Ichthyologists and Herpetologists (available at:http//www.asih.org/publications), and approved by SISBIO/Institute Chico Mendes de Conservação da Biodiversidade as a condition for the concession license. Specimens were collected in the municipality of Água Doce, Santa Catarina state, southern Brazil (26°35′59.9″S; 51°34′39.4″W; 1330 m above sea level) (Figure 1). Three adult males were collected on 8 January 2009 and 10 adult males on 5 January 2012. These frogs were found in two ponds subjected to anthropogenic impacts; these were separated by an unpaved road (the SC-452 highway). The area is within the Atlantic Forest biome and containes a mosaic of *Araucaria* forests and patches of natural grassland, with distinct swampy areas and patches of mixed rainforest [15]. The natural grassland formations of the highland plateaus of southern Brazil plateau are relicts of the drier and colder climates of the Pleistocene [15]–[17], which were mostly replaced by the subsequent expansion of *Araucaria* forest in the Holocene [18]. Água Doce municipality is located in the transitional zone of the high elevation grasslands known as the Campos de Palmas.

The regional climate is classified as Cfb (temperate) according to the Köppen-Geiger system. Mean annual temperature in this region is 10°C, with mean annual precipitation of 1500–200 mm [19]. The vegetation is predominantly herbaceous at the ponds where the specimens were collected, and composed primarily of plants of the Asteraceae, Cyperaceae and Poaceae families.

### Isolation, amplification, and sequencing of DNA

Genomic DNA was extracted from liver or muscle tissue and stored at −70°C in the tissue bank of the Departamento de Biologia Estrutural e Funcional of the Universidade Estadual de Campinas (UNICAMP), in São Paulo state, Brazil, using the TNES method, as applied by Bruschi et al. (2012). The mitochondrial 12S rDNA, tRNA-Val, and 16S ribosomal genes were amplified using the primers MVZ 59(L), MVZ 50(H), 12L13, Titus I (H), Hedges16L2a, Hedges16H10, 16Sar-L, and 16Sbr-H (for primer sequences, see reference [20]). The amplified PCR products were purified using Exonuclease I (10 units) and SAP (1 unit), with a 45-min incubation at 37°C and a 10-min denaturation at 85°C, then used directly as templates for sequencing in an automatic ABI/Prism DNA sequencer (Applied Biosystems, Foster City, CA, USA) with the BigDye Terminator kit (Applied Biosystems, Foster City, CA, USA), as recommended by the manufacturer. The DNA samples were sequenced bi-directionally and edited in Bioedit version 7.0.1 (http://www.mbio.ncsu.edu/BioEdit/bioedit.html) [21].

### Analysis of the molecular data

The initial sequence alignment was conducted for each gene separately, using CLUSTALW [22] in Bioedit, version 7.0.1 (http://www.mbio.ncsu.edu/BioEdit/bioedit.html). For each gene, the initial alignment was evaluated using four different gap penalties (5, 10, 15 and 20), and gap length was maintained constant (0.60) to identify regions of ambiguous homology [23]. The regions presenting ambiguous homologies were excluded for our phylogenetic inferences.

The phylogenetic relationships among the species were inferred from the concatenated matrix of the mitochondrial DNA 12S, tRNAval, and 16S rDNA sequences. We selected *Phyllomedusa* sequences available in the GenBank database, encompassing 90% of the species currently recognized for this genus [1]. The outgroup was *Agalychnis granulosa*, which was chosen based on the topology reported by Faivovich et al. [2]. A complete description of the species, sequences and GenBank accession numbers is provided in the Supporting Information (Table S1). Phylogenetic trees were constructed using Bayesian inference and the Maximum Parsimony method. Bayesian inference was based on a Markov chain Monte Carlo (MCMC) analysis conducted in MrBayes 3.1.2 [24] with two independent runs, each with four chains and sampling every 1000 generations for 6 million generations. An adequate burn-in (the first 25% trees were excluded) was determined by examining a plot of the likelihood scores of the heated chain for convergence and stationarity. The evolutionary model most appropriate for each gene was selected by MrMODELTEST [25] using the Akaike Information Criterion (AIC). The trees were sampled every 100 generations, excluding the first 25% of the trees as burn-in, determined by examining a plot of the likelihood scores the heated chain for convergence and stationarity. Tracer software version 1.5 [26] was used to confirm the quality of the parameters of the Bayesian inferences.

The Maximum Parsimony criterion was implemented in TNT v1.1 software [27] using a heuristic search method with tree bisection-reconnection (TBR) swapping and 100 random additional replicates. The bootstrap values of the branches inferred in this analysis were calculated with 1000 non-parametric pseudoreplicates.

Finally, the number of base substitutions per site among the sequences of the species of the *P. hypochondrialis* group was calculated using the maximum composite likelihood model [28] implemented in MEGA5 [29]. Gaps and missing data were eliminated in this analysis, and all parameters were at the default settings.

### Cytogenetic analysis

Ten male individuals were studied by cytogenetic methods (UFMG 13353–13362). Metaphase cells were obtained from the intestines and testes of animals that were previously treated with 2% colchicine, following procedures modified from King and Rofe [30] and Schmid [31]. Prior to the removal of the intestine and testes, the animals were deeply anesthetized following the recommendations of the Herpetological Animal Care and Use Committee (HACC) of the American Society of Ichthyologists and Herpetologists.

Cell suspensions were dripped onto clean slides and stored at −20°C. The chromosomes were stained with 10% Giemsa and silver stained using the Ag–NOR method according to Howel and Black [32], in addition to being C-banded as in Sumner [33], with some modifications. To better visualize the heterochromatin, the chromosomes were stained with the fluorochromes AT-specific DAPI (4′, 6-diamidino-2-phenylindole) and GC-specific Mytramicim (MM) sequentially C-banding. To confirm their number and positions, the rDNA sites was detected using fluorescence *in situ* hybridization (FISH) as in Viegas-Péquignot [34] using the HM123 probe [35]. The metaphases were photographed under an Olympus microscope and analyzed using Image Pro-Plus software, version 4 (Media Cybernetics, Bethesda, MD, USA). The chromosomes were measured, and the centromere index (CI), relative length (RL), and centromere ratio (CR) were estimated. The chromosomes were ranked and classified according to the scheme of Green and Sessions [36].

### Morphological Analyses

The specimens examined for the description of the new species, including those of other taxa used for comparison are deposited in the following Brazilian institutions: CFBH (Célio F. B. Haddad amphibian collection, Universidade Estadual Paulista, Rio Claro, São Paulo state); CAUC (Coleção de Anfíbios da Universidade Comunitária da Região de Chapecó, Chapecó, Santa Catarina state); UFMG (Coleção Herpetológica da Universidade Federal de Minas Gerais, Belo Horizonte, Minas Gerais state), and MNRJ (Museu Nacional, Rio de Janeiro, Rio de Janeiro state) (see list of examined specimens in Appendix S1). The webbing formula notation followed Savage and Heyer [37], as modified by Myers and Duellman [38].

Measurements of the adult specimens were conducted using a Mytutoyo digital caliper to the nearest 0.01 mm: snout-vent length (SVL), head length (HL), head width (HW), eye diameter (ED), tympanum diameter (TD), eye-to-nostril distance (END), nostril-to-tip of snout distance (NSD), internarial distance (IND), upper eyelid width (UEW), interorbital distance (IOD), tibia length (TL) and foot length (FL) [39]; the distance between the anterior margins of the eyes (AMD) [40]; forearm length (FAL) [41]; hand length (HAL), thigh length (THL) and tarsal length (TAL), following Heyer et al [42]; as well as the disk diameter of the third finger (3FD) and the fourth toe (4TD) [43].

### Nomenclatural Acts

The electronic edition of this article adheres to the requirements of the amended International Code of Zoological Nomenclature, and the new names contained herein are therefore available under that Code in the electronic edition of this article. The published work and the nomenclatural descriptions it contains have been registered in ZooBank, the online registration system for the ICZN. The ZooBank LSIDs (Life Science Identifiers) can be obtained and the associated information viewed through any standard web browser by appending the LSID to the prefix “http://zoobank.org/”. The LSID for this publication is urn:lsid:zoobank.org:pub:9A74B7B3-32C8-4BAA-86BC-9247128B782E. The electronic edition of this study was published in a journal with an ISSN, has been archived, and is available from the following digital repositories: PubMed Central, LOCKSS.

## Results

### Phylogenetic Inferences

The combined matrix consisted of 2141 bps. In the Bayesian inference, the GTR+G+I model was identified as being the most appropriate evolutionary model for the data set. The topology indicated similar relationships among the species of the genus to those described by Faivovich et al [2] and Bruschi et al. [8]. Two clades were observed within the current arrangement of the *P. hypochondrialis* group: the first clade included the species *P. palliata*, *P. hypochondrialis*, *P. nordestina* and *P. azurea*, while *P. megacephala*, *P. rohdei*, “*P. rhodei”*, *P. centralis*, *P. oreades* and haplotypes of *Phyllomedusa* sp. n. from Água Doce, Santa Catarina State, constituted the second clade. Similar relationships were observed in the MP analyses, though different support values were obtained.

**Figure 2:**
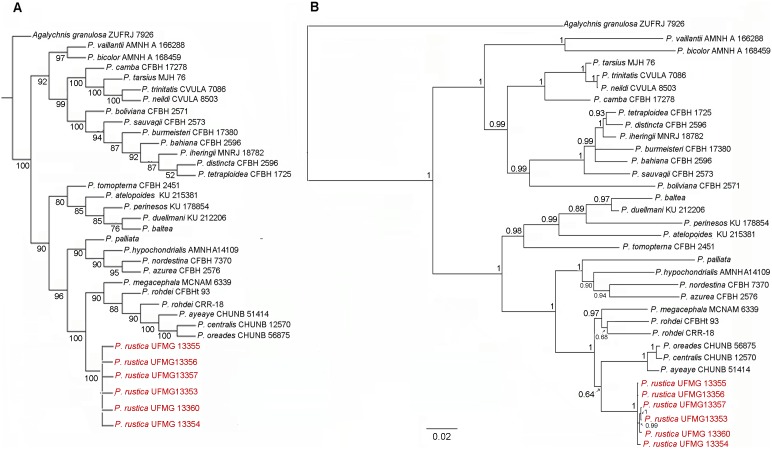
Phylogenetic relationships of the genus *Phyllomedusa* based on 2141 bps of the mitochondrial 12S rDNA, tRNA-val and 16S rDNA mitochondrial genes. (**A**) Strict consensus cladogram inferred using the maximum parsimony (MP) criterion, implemented in the TNT program. Numbers adjacent to the nodes indicate bootstrap values. (**B**) Topology inferred from the Bayesian analysis based on the GTR+R+I model. Bayesian posterior probabilities are shown at each node. Scale bar represents the number of substitutions per site.

The parsimony and Bayesian trees detected a minor conflict in the internal relationships among the species within the second subclade (Figure 2A–B). In the Parsimony topology, *Phyllomedusa* sp. n. is monophyletic and is a sister group to all of the species of the second clade, with strong bootstrap support (Figure 2A), whereas in the Bayesian inference (Figure 2B), *Phyllomedusa* sp. n. is grouped with *P. ayeaye*, *P. centralis* and *P. oreades* and this subclade is the sister group of remaining species. However, this arrangement is weakly supported by the posterior probability values.

Considering the genetic divergence in the 1335 positions that make up the final dataset of 12S, t-RNA-val and 16S mitochondrial fragments, the level of sequence divergence between *Phyllomedusa* sp. n. and other species of the *P. hypochondrialis* group ranged from 6% to 12% (Table 1). Within the second clade of the *P. hypochondrialis* group, divergence levels varied from 6% to 11%. The reduced divergence between the sequences of *P. centralis*, *P. oreades*, and *P. ayeaye* (1–2%) has been observed previously in cytochrome b sequences [2].

**Table 1:** Uncorrected pairwise distances between the 12S, tRNA-val and 16S mitochondrial sequences of the species of the *Phyllomedusa hypochondrialis* group.

Species	1	2	3	4	5	6	7	8	9	10
**1**- *P. palliata*	-									
**2-** *P. hypochondrialis*	0.11	-								
**3-** *P. nordestina*	0.11	0.10	-							
**4-** *P. azurea*	0.12	0.11	0.10	-						
**5-** *P. megacephala*	0.11	0.10	0.10	0.11	-					
**6-** *P. rohdei* CFBHt 93	0.12	0.11	0.11	0.12	0.05	-				
**7-** *P. rohdei* CRR-18	0.11	0.11	0.11	0.12	0.06	0.06	-			
**8-** *P. centralis*	0.13	0.11	0.11	0.12	0.07	0.07	0.08	-		
**9-** *P. oreades*	0.13	0.11	0.11	0.12	0.07	0.07	0.07	0.01	-	
**10**- *P. ayeaye*	0.12	0.11	0.10	0.12	0.07	0.06	0.07	0.02	0.01	-
**11-** *Phyllomedusa rustica* sp. n.	**0.11**	**0.10**	**0.11**	**0.11**	**0.05**	**0.06**	**0.06**	**0.06**	**0.06**	**0.06**

### Cytogenetic Analysis

**Figure 3:**
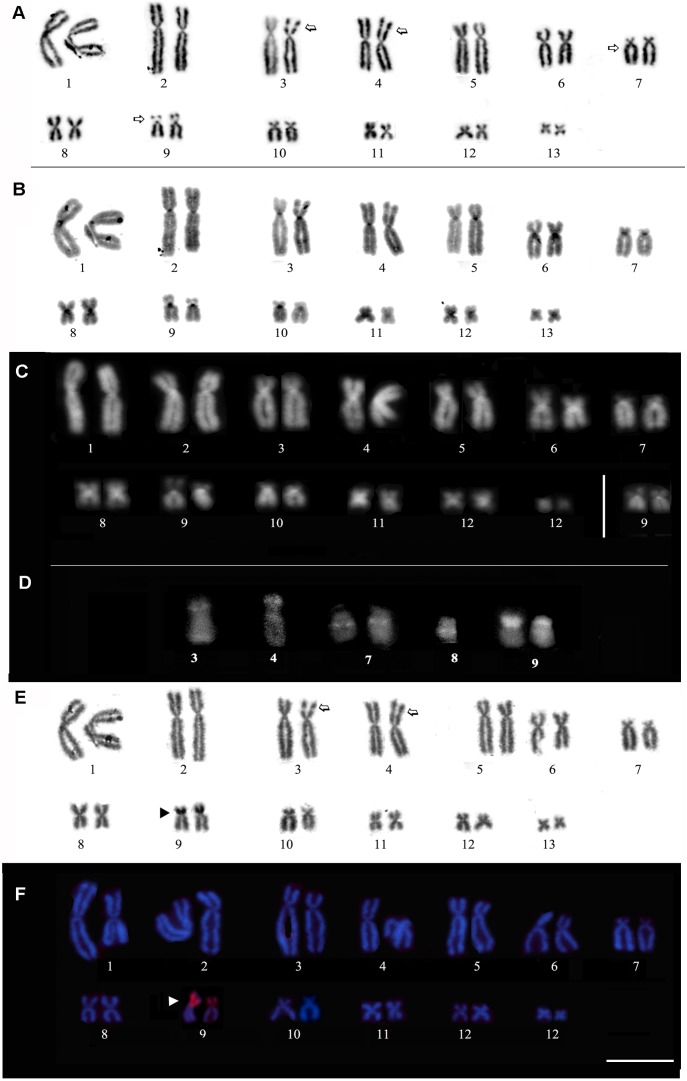
Karyotype of *Phyllomedusa rustica* sp. n. based on Giemsa staining (A), C-banding (B), DAPI staining after C-banding (C), and chromosomes submitted to Mytramicim (MM) staining after C-banding, silver impregnation via the Ag-NOR method (D) and FISH experiments with a nucleolar 28S rDNA probe. Arrows in (**A**) indicate secondary constrictions. The inset in (**C**) shows the homomorphic condition of homologue pair 9. In **D**, the chromosome pairs submitted to MM staining exhibit brilliant fluorescence, coinciding with the secondary constrictions detected by Giemsa-staining. The arrowheads indicate NOR sites in (**E**) and (**F**).

The analyzed specimens all presented a diploid number of 2n = 26 chromosomes. The karyotype consisted of four metacentric (1, 4, 8, 11), six submetacentric (2, 3, 5, 6, 12, and 13) and three subtelocentric pairs (7, 9 and 10) (Figure 3). Secondary constrictions were observed in the short arms of pair 9 and in one of the homologs in pairs 3 and 4, both in the subterminal short arms (Figure 3A). In some metaphases, secondary constrictions were observed through conventional Giemsa staining of the long arms of pair 7, in the pericentromeric region. The heterochromatin pattern revealed a C-positive block at the centromeric position in all pairs (Figure 3B). C-bands were evident in the pericentromeric region of the long arm of pair 6. When DAPI staining was analyzed (Figure 3C), DAPI-positive bands were observed in the centromeric regions of all chromosomes, presenting a similar pattern to that found by C-banding. MM staining revealed brilliant fluorescence in the same regions as the secondary constrictions that were detected by conventional staining (Figure 3D).

The Ag-NOR and FISH techniques conducted using an rDNA probe revealed that the new species has a single NOR pair located in the subterminal region on chromosome pair 9 (Figure 3E and F). In this chromosome pair, the NOR was detected in the secondary constriction observed through Giemsa and MM-positive banding. The NOR sites presented size heteromorphism in the three specimens analyzed.

#### *Phyllomedusa rustica* sp. n

urn:lsid:zoobank.org:act:DD2C2754-AD90-4E63-96E4-C6D61972A4B1.

*Phyllomedusa azurea* **–** Lucas, Fortes and Garcia 2010.

#### Holotype (Figure 4 and 5)

UFMG 13360, an adult male from Água Doce municipality (26°35′59.9″ S, 51°34′39.4″ W; 1330 m above sea level), Santa Catarina state, Brazil, collected by Elaine M. Lucas, Daniel Bruschi, and Veluma Debastiani, on 5 January 2012.

#### Paratopotypes

Nine adult males, UFMG 13353–13359 and 13361–13362, collected together with the holotype; three adult males, UFMG 1585 (Ex UFMG 3222) and CAUC 0864–0865, collected by Elaine M. Lucas, on 8 January 2009, at the type locality.

#### Type-locality

Água Doce municipality, Santa Catarina state, southern Brazil.

#### Diagnosis

A small species of *Phyllomedusa* belonging to the *P. hypochondrialis* species group, diagnosed based on the following combination of characters: (1) small to medium size (SVL 33.9–37.1 mm in males); (2) body moderately slender; (3) snout nearly rounded to truncate in dorsal view, vertical to obtuse in profile; (4) flanks and hidden areas of the arms and legs cream to reddish orange with black reticulations; (5) distinct pattern of reddish orange cells encircled by black or dark blue coloring on the concealed surfaces of the limbs; (6) upper lip lacking a reticulate pattern; (7) discreet reticulated pattern on the edge of the lower eyelids; (8) throat and belly whitish to creamy orange, slightly reticulated; (9) dorsal surfaces uniformly green, without spots; (10) dorsal surfaces smooth without granules; (11) translucent palpebral membrane slightly reticulated.

*Phyllomedusa rustica* sp. n. differs from *P. azurea, P. centralis, P. hypochondrialis* and *P. nordestina* by the distinct pattern of reddish orange small blotches encircled by black or dark blue coloring on the concealed surfaces of the limbs and by the slightly reticulate pattern on the border of the eyelids, and the ventral surfaces of the body (*P. centralis, P. hypochondrialis* and *P. nordestina* are characterized by a pattern of black bars or stripes on the red-orange bottom and an absence of reticulation on the ventral surfaces of the body); from *P. oreades* and *P. ayeaye* based on the absence of a reticulated pattern on the margins of the upper lip (present in these species) and less conspicuous reticulation on the ventral regions of the limbs, jaw, and body (conspicuous in these species); from *P. megacephala* by the presence of a discreet reticulate pattern on the border of the eyelids and the ventral surfaces of the body (absent in this species); from *P. rohdei* and *P. palliata* based on the absence of a whitish stripe along the lateral part of head (present in these species; see [41] regarding *P. palliata*).

#### Description of the holotype

**Figure 4:**
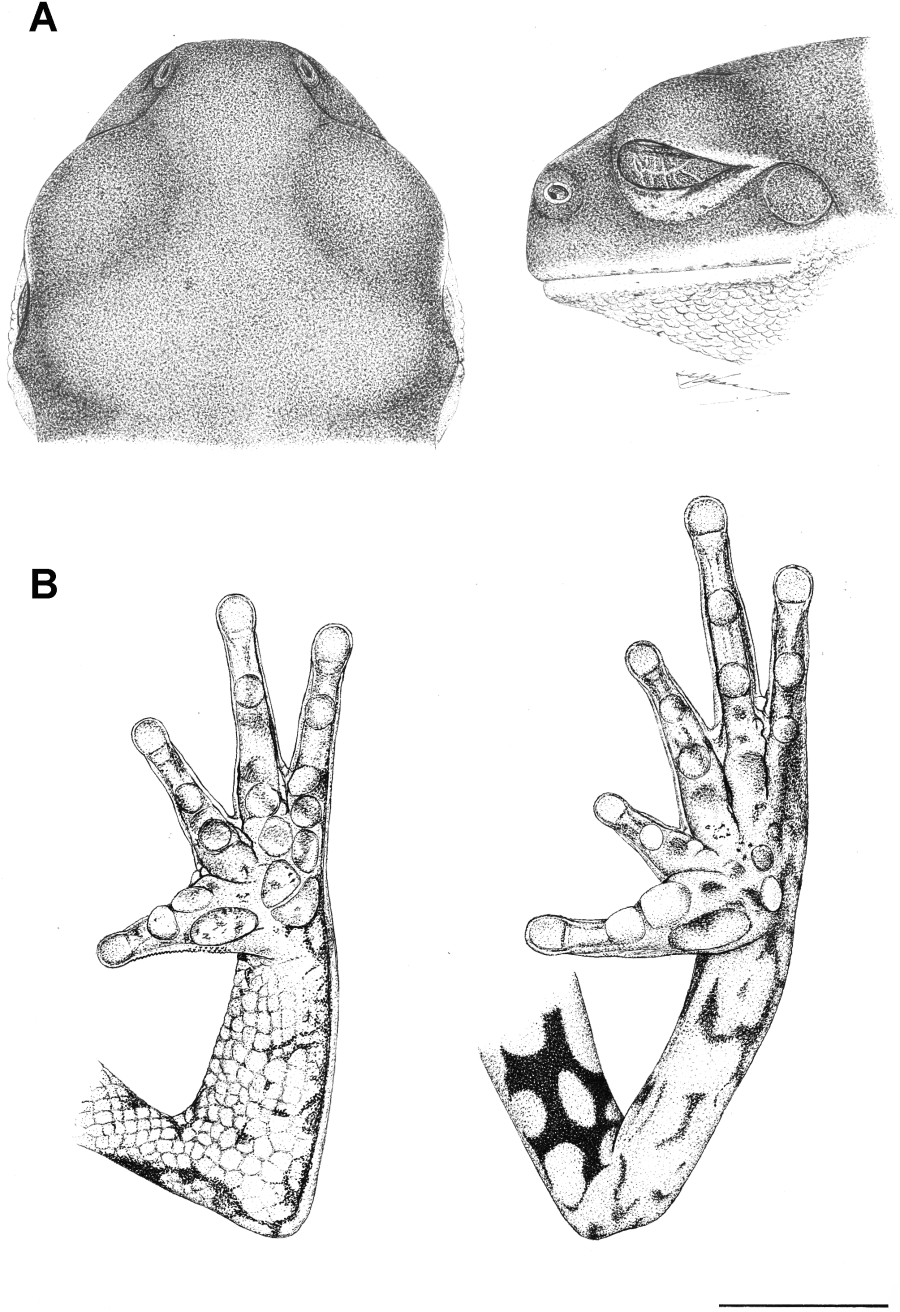
Holotype of *Phyllomedusa rustica* sp. n. (UFMG 13360), adult specimen: (A) dorsal and lateral views of head and (B) palmar (left) and plantar (right) views. Scale Bar = 5 mm.

**Figure 5:**
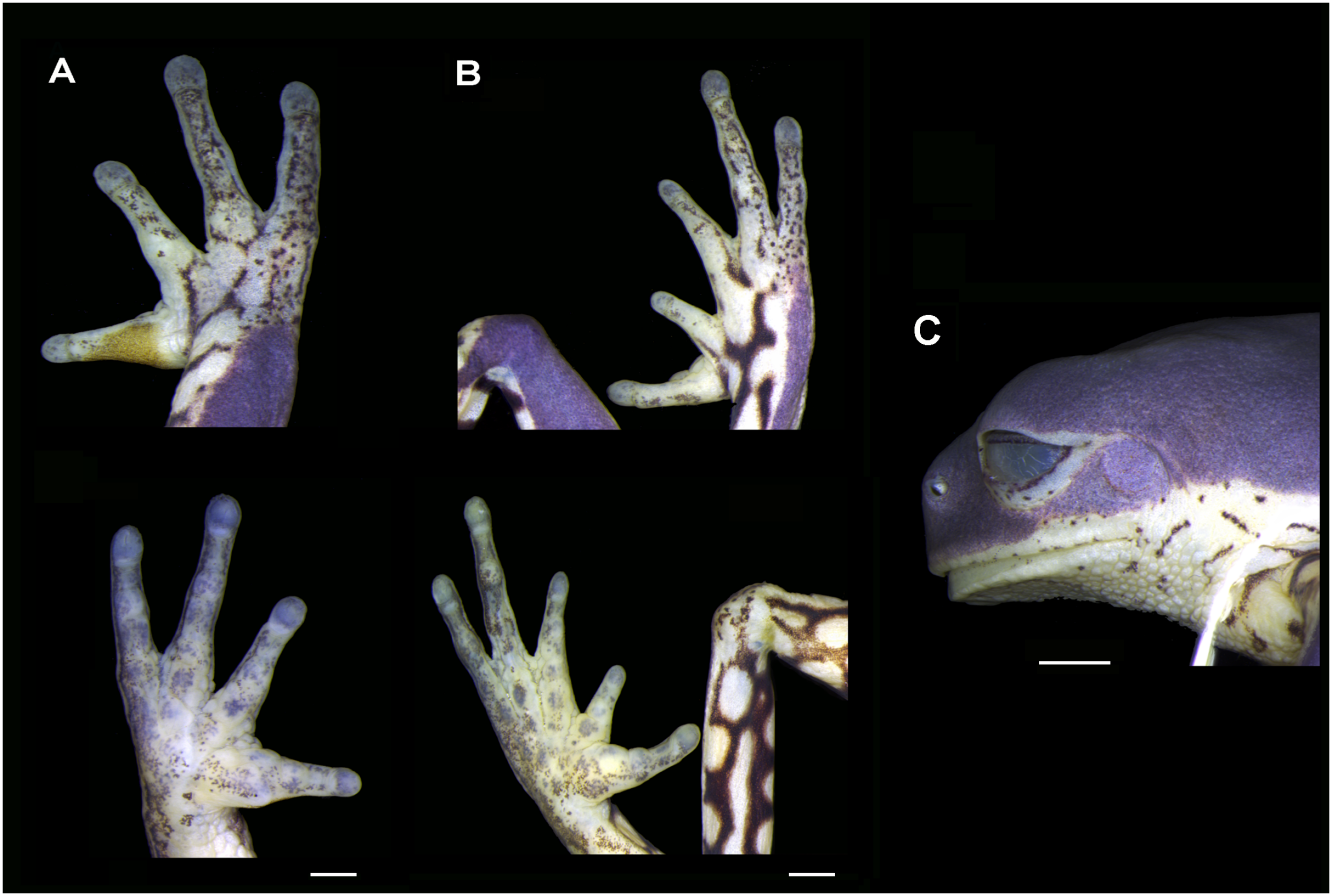
Holotype of *Phyllomedusa rustica* sp. n. (UFMG 13360), adult specimen: (A) dorsal (top) and ventral (bottom) views of hand, (B) dorsal (top) and ventral (bottom) views of foot (C) lateral view of the head. Scale bar = 2 mm.

General aspect slender; head slightly wider than long, SVL approximately three times the head width; snout nearly round to truncate in dorsal view (Figure 4) and vertical-to-slightly obtuse in lateral view (Figure 4 and 5); loreal region concave; nostrils small, circular and anterolaterally directed, near the tip of snout; eyes large, circular and lateral-frontally positioned; vertically elliptical pupil; translucent eyelid membrane slightly reticulated; tympanum nearly circular, with a diameter equal to approximately half of the eye diameter, annulus undefined at the superior border; faint supratympanic fold present; parotoids undistinguished; no vocal slits or external vocal sac; tongue large, piriform, free posteriorly; choanae small, nearly circular; vomerine teeth not visible externally; choanae small, largely separated; arm slender; forearm robust; no finger webbing; comparative finger lengths I<II<IV<III; finger tips not expanded, smaller than the tympanum diameter; palmar callosities large, circular (Figure 4); inner and outer metacarpal tubercles undifferentiated; subarticular tubercles well developed, globose; finger I enlarged at the base, opposed to the other fingers; nuptial pad of horny asperities evident in males; legs short, moderately robust; thigh length slightly longer than tibia length and both shorter than the tarsus-foot length; sum of the thigh and tibia lengths, slightly greater than 80% of SVL; calcar appendage and tarsal fold absent; foot (Figures 4 and 5) with slender toes, not webbed or fringed, with apical disks poorly developed, globose, smaller than the tympanum diameter; comparative toe length II<I = III<V<IV; toes I and II opposed to the others; subarticular and supernumerary tubercles single, large, round (Figure 4), inner and outer metatarsal tubercles undifferentiated; no toe webbing; dorsal skin smooth; ventral skin on the belly and throat granular; ventral surface of thigh granular anteriorly and smooth posteriorly; cloacal region moderately glandulose; cloacal opening not modified. The measurements and proportions of the body parts of the holotype are presented in Tables 2 and 3, respectively.

**Table 2:** Mean ± standard-deviation (SD), and range of the measurements (in mm) of the males of type series of *Phyllomedusa rustica* sp. n. and the holotype (see Material and methods for abbreviations).

Characters	Type series of *Phyllomedusa rustica* sp. n.(n = 11)	Holotype UFMG 13360
**SVL**	35.46±1.16 (33.93–37.09)	35.53
**HL**	10.66±0.47 (9.86–11.4)	11.4
**HW**	11.85±0.41 (11.17–12.61)	12.16
**ED**	3.75±0.36 (3.17–4.21)	3.54
**TD**	2.17±0.18 (1.86–2.36)	2.32
**END**	2.71±0.22 (2.40–3.10)	2.4
**NSD**	1.84±0.29 (1.32–2.19)	1.85
**IND**	3.62±0.34 (2.96–4.14)	3.38
**UEW**	3.23±0.27 (2.80–3.66)	3.18
**AMD**	6.96±0.35 (6.44–7.82)	6.94
**IOD**	4.58±0.38 (4.16–5.45)	4.31
**FAL**	8.61±0.91 (6.54–9.52)	6.54
**HAL**	9.88±0.52 (8.93–10.59)	9.9
**3FD**	0.98±0.12 (0.81–1.25)	0.83
**THL**	15.33±0.78 (14.21–16.54)	15.31
**TL**	14.10±0.60 (13.02–14.94)	13.64
**TAL**	12.73±0.59 (11.96–13.61)	12.29
**FL**	22.44±1.08 (20.72–23.75)	22.14
**4TD**	1.07±0.17 (0.81–1.27)	1.07

**Table 3:** Mean ± standard deviation (SD), and range in parentheses of the ratios of the body parts in the type series of *Phyllomedusa rustica* sp. n. and the holotype (see Material and methods for abbreviations).

Rations	Type series of *Phyllomedusa rustica* sp. n.(n = 11)	Holotype UFMG 13360
**HL/SVL**	0,30±0,01 (0.28–0.32)	0.32
**HW/SVL**	0,33+0,01 (0.32–0.35)	0.34
**FAL/SVL**	0,24±0,02 (0.18–0.27)	0.18
**HAL/SVL**	0,28+0,01 (0.26–0.30)	0.28
**THL/SVL**	0,43±0,02(0.40–0.47)	0.43
**TL/SVL**	0,40±0,01 (0.38–0.43)	0.38
**TAL/SVL**	0,36±0,01 (0.35–0.38)	0.35
**FL/SVL**	0,63±0,02 (0.59–0.68)	0.62
**HW/HL**	1,11±0,04 (1.03–1.16)	1.07
**ED/HL**	0,36±0,02 (0.31–0.39)	0.35
**TD/HL**	0,20±0,02 (0.16–0.23)	0.18
**END/HL**	0,26±0,02 (0.21–0.29)	0.21
**NSD/HL**	0,05±0,01 (0.04–0.06)	0.05
**IND/HL**	0,10±0,01 (0.09–0.12)	0.10
**UEW/HL**	0,30±0,02 (0.26–0.34)	0.28
**AMD/HL**	0,65±0,02 (0.61–0.69)	0.61
**TD/ED**	0,57±0,07 (0.44–0.74)	0.52
**3FD/TD**	0,10±0,01 (0.08–0.12)	0.08
**4TD/3FD**	1,10±0,14 (0.90–1.29)	1.29

#### Coloration in life

**Figure 6:**
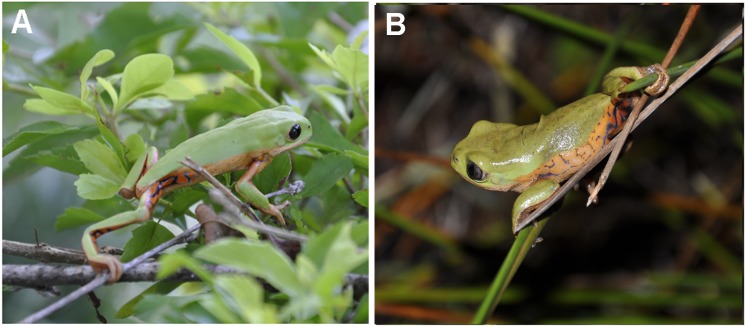
Paratypes of *Phyllomedusa rustica* sp. n. (A) Coloration pattern in the flank regions; (B) individual during call activity perched near the ground in herbaceous vegetation at the margin of a pond.

Dorsum and dorsal surfaces of the forearm, shank, tarsus, and foot light green, olive or brownish green, varying within an individual (Figure 6); flanks, hidden areas of the arms and legs, and dorsal surface of the hands and feet cream-orange with black or dark blue reticulations; the dorsal surface of the thigh presents only a narrow green strip that tapers toward the groin without contacting it; axial and inguinal areas with black or dark blue reticulation, encircling large vivid reddish orange blotches; tympanic membrane green, similar to the dorsum; posterior border of the maxillae and margin of the lower eyelid with a fine light stripe and discreet dark reticulations on the lower eyelid; throat, belly and ventral surface of thighs whitish, lightly reticulated; iris silver with fine dark reticulation; pupil black; translucent palpebral membrane discretely reticulated.

#### Color in preservative

Dorsal surfaces of the body, forearms, tarsus and shank bluish gray; blackish purple reticulations on the flanks and hidden parts of the thigh and ventral surface of the shank; reticulated pattern of flanks increasing in size from the angle of the mouth to the groin; belly and chest discretely reticulated; lateral groin reticulations encircling large whitish mottling. A narrow, light stripe with fine dark reticulations on the posterior is found on the margin of the upper jaw and the margin of the lower eyelid.

#### Variation

Variation was found among individuals in body size (Table 2) and proportions (Table 3). In the paratypes UFMG 13358, 13360 and 13362, most of the ventral region is reticulated.

#### Natural history

Male *Phyllomedusa rustica* sp. n. were observed perched near the ground during calling activity, frequently in association with herbaceous vegetation at the margins of ponds (Figure 6). When we handled the specimens, the animals contracted their bodies [44], with the belly, arms, and legs upturned, remaining motionless (Figure 5). We observed the eight other anuran species in the same pond: *Dendropsophus minutus, Hypsiboas leptolineatus, Hypsiboas pulchellus, Sphaenorhynchus surdus, Leptodactylus latrans, Leptodactylus plaumanni, Physalaemus cuvieri* and *Physalaemus* aff. *gracilis*.

#### Distribution

Known only from the type locality, in the grasslands of southern Brazil, in the municipality of Água Doce in the state of Santa Catarina.

#### Etymology

The epithet *rustica* originates from the Latin *rusticus* and is used to indicate the characteristics of the fields where this species is found (open fields). The epithet is as an adjective.

## Discussion

Specimens of a disjunct population of *Phyllomedusa* from southern Brazil were analyzed through phylogenetic inference, as well as morphological and comparative chromosomal analyses, which allowed us to recognize a new species of the *P. hypochondrialis* group, which we have described here and named *Phyllomedusa rustica* sp. n.

This population was first reported by Lucas et al. [14], who concluded that this was the first population of *P. azurea* to be found outside the savanna biome of South America. Despite this identification, the authors observed the variable morphological characteristics in this population and suggested the existence of a *P. azurea* species complex [14]. Coloration patterns have been used universally for the delimitation of amphibian species, including those of the *Phyllomedusa hypochondrialis* group [45]–[46]. However, these characteristics do not appear to be consistently useful for the diagnosis of the taxa of this group, and are unable to discriminate reliably groups of species such as *P. hypochondrialis*, *P. azurea*, and *P. nordestina* [2], despite the fact that coloration patterns are the main diagnostic criteria used to differentiate these species [3]. The discrimination of *P. araguari* and *P. oreades* [47], for example, and *P. itacolomi* and *P. ayeaye* [48], has proven similarly problematic. In amphibians, DNA markers have been helpful for the recognition of cryptic lineages [9], [49]–[50] and are especially important in groups which lack clear morphological differences [49], [51]. In the present study, the topology obtained from the molecular analyses was fundamental for the recognition of the new species. A similar topology was reported previously [8], based on the comparison of populations from Brazil assigned to *P. hypochondrialis* and *P. azurea*, highlighting the paraphyletic position of the Água Doce population in relation to *P. azurea* from the Pantanal and Chaco formations. The topology obtained in the present study provides strong evidence to justify our taxonomical decision to recognize *Phyllomedusa rustica* sp. n.

The karyotype of *Phyllomedusa rustica* sp. n. had the same diploid number (2n = 26) and conserved chromosome morphology as all other representatives of the genus [8], [52]–[57]. The NOR-bearing chromosomes in *Phyllomedusa rustica* sp. n. are the small submetacentric chromosomes of pair 9 (subtelomeric region), as observed in *P. nordestina* [55], *P. rhodei* [55] and *P. ayeaye* (Bruschi, unpublished data). In the karyotype of the new species, a bright MM fluorescence pattern was observed in the region of the secondary constrictions using conventional staining. Despite this characteristic being consistently associated with NOR sites, with the exception of the NOR on pair 9, no hybridization signals were detected with the 28S rDNA probe in any of the other secondary constrictions (chromosomes 3, 4, 7 and 8). In these chromosomes, the MM-positive marks may be associated with the composition of this class of chromatin, with repetitive sequences rich in GC bases occurring in the chromosomal constrictions. The observed C-patterns were mainly centromeric, which is a common characteristic of the karyotypes of the *P. hypochondrialis* group [8], [55].

*Phyllomedusa rustica* sp. n. was collected at altitude of 1330 m above sea level, within the Atlantic Forest formation of southern Brazil. *Phyllodemusa rohdei* is another species of the same subclade that is also found in the Atlantic Forest. All other species of this clade inhabit plateaus and mountainous regions in the Cerrado savannas of central Brazil [2]. In southern Brazil, the Atlantic Forest biome includes grasslands on the high plateaus that form a forest mosaic in the northern half of Rio Grande do Sul and in the states of Santa Catarina and Paraná. The Água Doce region is included in the Atlantic Forest *sensu lato* [58], which is characterized by seasonal deciduous forest, forming a natural mosaic with grasslands and *Araucaria* forests in western Santa Catarina and Paraná states, along the upper Uruguay River, extending to the Ibicuí and Jacuí basins in the center of the state of Rio Grande do Sul.

Special efforts are needed to define the taxonomic arrangement of the second subclade of *P. hypochondrialis* group, to which *Phyllomedusa rustica* sp. n. belongs, due primarily to difficulties in taxonomic delimitation based only in morphological traits. One example of a taxonomic problem is provided by the different populations of *P. rhodei*, in which strong evidence of mitochondrial sequence divergence was detected [2], indicating the existence of cryptic diversity – at least two distinct species – among the frogs currently assigned to *P. rhodei*. Faivovich et al. [59] corroborated this suggestion based on anatomical differences between populations from the type locality (Rio de Janeiro, Brazil) and Espírito Santo, Brazil. However, this specific question was not addressed within the scope of this work, and we can only note that the resolution of taxonomic questions in this subclade is important and that the identification of new lineages will contribute to a better understanding of their evolutionary history.

### Underestimation of species diversity and conservation of Atlantic Forest frogs

The recognition of the new species has important implications for the development of conservation strategies in southern Brazil, as well as for the understanding of ecological and evolutionary patterns, in particular the biological diversification of the Atlantic Forest. The description of this new species further reinforces the conclusion that the true diversity of the amphibian fauna of the Atlantic Forest biome has been underestimated substantially [60]–[61]. In recent years, the application of molecular approaches has contributed to the fine-scale taxonomic re-evaluation of many amphibian groups, revealing many new cryptic lineages in the Atlantic Forest (e.g. see references [9], [62]–[65]). Indeed, this biome has proven to be a biodiversity hotspot with a high level of species endemism in many vertebrate groups [66]–[68], and the understanding of the mechanisms underlying the biological diversification of this biome is of enormous interest to herpetologists [68]–[70].

Despite its accentuated species richness and endemism, the Atlantic Forest has suffered high rates of habitat loss throughout its recent history [71]. This biome originally covered an area of over 1,300,000 km^2^ of eastern and southern Brazil, extending as far west as Paraguay and Argentina. This area has now been reduced to less than 12% of its original extent, most of which is distributed in small and isolated fragments [71]. Protected areas account for only 1.6% of this domain and do not cover the different vegetation types adequately [71]–[72]. The type locality of *Phyllomedusa rustica* sp. n., is located within a region classified as “extremely high priority” for conservation actions in the National Plan for the Conservation of the Herpetofauna of Southern Brazil - PAN Herpetofauna do Sul [73]. In spite of this, the natural local grasslands are still being converted into *Pinus* plantations or farmland [74], leading to profound modifications of the structure and composition of local ecological communities, and impacts on the biodiversity of the region. The description of a new Atlantic Forest species contributes to the understanding of the evolutionary history of the biota of this formation [75] and the phylogenetic diversity of its different ecosystems [76]–[77]. A better understanding of a region’s biodiversity [78], the life-history traits of its species [79]–[81] and their evolutionary history [76]–[77], [79], [82]–[83] may be especially important for the identification of priority areas in conservation planning.

## Supporting Information

Table S1 (DOC)

Appendix S1 (DOCX)
