# Supplementary material for: Molecular and Morphological Evidence Reveals a New Species in the Phyllomedusa hypochondrialis Group (Hylidae, Phyllomedusinae) from the Atlantic Forest of the Highlands of Southern Brazil
Source: PLoS One. 2014 Aug 20;9(8):e105608. doi: 10.1371/journal.pone.0105608 (PMC4139387; doi:10.1371/journal.pone.0105608)
Supplement: Table S1 — (DOC) [file pone.0105608.s001.doc]

**Table S1:** Details (species, voucher number, sample locality, accession number and authors) of the sequences obtained in this work and from GenBank used for phylogenetic inferences.

| **Species** | **Voucher** | **Locality** | **12S-tRNAval-16S** | **Reference** |
| --- | --- | --- | --- | --- |
| *Agalychnis granulosa* | ZUFRJ 7926 | Brazil, Pernambuco, Jaqueira | AY843687 | Faivovich et al. 2005 |
| *Phyllomedusa ayeaye* | CHUNB 51414 | Brazil, Minas Gerais, Poços de Caldas | GQ366245 | Faivovich et al. 2010 |
| *Phyllomedusa atelopoides* | KU215381 | Peru, Madre de Dios | AY819413 | Wiens et al., 2005 |
| *Phyllomedusa azurea* | CFBH 2576 | Brazil, Mato Grosso, Corumbá | GQ366248 | Faivovich et al. 2010 |
| *Phyllomedusa bahiana* | CFBH 2596 | Brazil , Sergipe, Areia Branca | GQ366251 | Faivovich et al. 2010 |
| *Phyllomedusa baltea* | To be deposited in SMNS | Peru, Pasco, Santa Cruz | GQ366252 | Faivovich et al. 2010 |
| *Phyllomedusa bicolor* | AMNHA-168459 | Pet (Germany) | AY843723 | Faivovich et al., 2005 |
| *Phyllomedusa boliviana* | CFBH 2571 | Brazil , Mato Grosso, Corumbá | GQ366253 | Faivovich et al. 2010 |
| *Phyllomedusa burmeisteri* | CFBH 17360 | Brazil , Minas Gerais, Furnas | GQ366256 | Faivovich et al. 2010 |
| *Phyllomedusa camba* | CFBH 17278 | Brazil , Rondônia, Ministra Andreazza | GQ366259 | Faivovich et al. 2010 |
| *Phyllomedusa centralis* | CHUNB 12570 | Brazil, Mato Grosso, Chapada dos Guimarães | GQ366260 | Faivovich et al. 2010 |
| *Phyllomedusa distincta* | CFBH 2658 | Brazil, Paraná, Guaratiba | GQ366262 | Faivovich et al. 2010 |
| *Phyllomedusa duellmani* | KU212206 | Peru, San Martin, Rioja | AY819414 | Wiens et al., 2005 |
| *Phyllomedusa hypochondrialis* | AMNH A-141109 | Guyana, Dubulay Ranch on the Berbice River | AY843724 | Faivovich et al., 2005 |
| *Phyllomedusa iheringii* | MNRJ 18782 | Brazil, Rio Grande do Sul, Santa Maria | GQ366264 | Faivovich et al. 2010 |
| *Phyllomedusa megacephala* | MCNAM 6339 | Brazil, Minas Gerais, Serra do Cipó | GQ366267 | Faivovich et al. 2010 |
| *Phyllomedusa neildi* | CVULA 6503 | Venezuela, Falcon, Municipio Petit | GQ366270 | Faivovich et al. 2010 |
| *Phyllomedusa nordestina* | CFBH 7330 | Brazil, Alagoas, Passos dos Camarajibe | GQ366271 | Faivovich et al. 2010 |
| *Phyllomedusa oreades* | CHUNB 56875 | Brazil, Goiás, Serra de Caldas | GQ366278 | Faivovich et al. 2010 |
| *Phyllomedusa palliata* | To be deposited in SMNS | Bolívia, Beni, Rurrenabaque | GQ366280 | Faivovich et al. 2010 |
| *Phyllomedusa perinesos* | KU178854 | Ecuador, Napo | GQ896278 | Wiens et al., 2010 |
| *Phyllomedusa “rohdei”* | CFBHt 93 | Brazil, São Paulo, Ubatuba | GQ366237 | Faivovich et al. 2010 |
| *Phyllomedusa “rohdei”* | CRR-18 | Brazil, Minas Gerais, Perdizes | GQ366240 | Faivovich et al. 2010 |
| *Phyllomedusa sauvagii* | CFBH 2573 | Brazil, Mato Grosso, Corumbá | GQ366281 | Faivovich et al. 2010 |
| *Phyllomedusa tarsius* | MJH 67 | Brazil, Amazonas, Reserva Ducke | AY843726 | Faivovich et al., 2005 |
| *Phyllomedusa tetraploidea* | CFBH 1725 | Brazil, São Paulo, Ribeirão Branco | GQ366285 | Faivovich et al. 2010 |
| *Phyllomedusa tomopterna* | CFBH 2451 | Amazonas, Manaus | GQ366286 | Faivovich et al. 2010 |
| *Phyllomedusa trinitatis* | CVULA7086 | Venezuela, Miranda | GQ366287 | Faivovich et al. 2010 |
| *Phyllomedusa vaillanti* | AMNH A- 166288 | Guyana, Berbice River | AY549363 | Faivovich et al., 2005 |
| *Phyllomedusa sp.* | UFMG 13353 | Brazil:Santa Catarina: Água Doce | KC520734 | Bruschi et al., 2013 |
| *Phyllomedusa sp.* | UFMG 13354 | Brazil:Santa Catarina: Água Doce | KC520705 | Bruschi et al., 2013 |
| *Phyllomedusa sp.* | UFMG 13355 | Brazil:Santa Catarina: Água Doce | KM206705 | Present study |
| *Phyllomedusa sp.* | UFMG 13356 | Brazil:Santa Catarina: Água Doce | KM206706 | Present study |
| *Phyllomedusa sp.* | UFMG 13357 | Brazil:Santa Catarina: Água Doce | KM206707 | Present study |
| *Phyllomedusa sp.* | UFMG 13360 | Brazil:Santa Catarina: Água Doce | KM206708 | Present study |

**Abreviation:** AMNH (American Museum of Natural History, USA); CFBH (Coleção Célio Fernando Baptista Haddad, Universidade Estadual Paulista, Brazil); CHUNB (Coleção Herpetológica Universidade Nacional de Brasília, Brazil); CRR (Camila R. Rabelo Field Series); CVULA (Colección de Vertebrados Universidad de los Andes, Venezuela); KU (The University of Kansas, Museum of Natural History, Lawrence, Kansas, USA); MACN (Museo Argentino de Ciencias Naturales ‘‘Bernardino Rivadavia’’, Argentina); MCNAM (Museu de Ciências Naturais, Pontifícia Universidade Católica de Minas Gerais, Brazil); MJH (Martin J. Henzel field series); MNRJ (Museu Nacional Universidade Federal do Rio de Janeiro, Brazil); UFMG (Coleção Herpetológica da Universidade Federal de Minas Gerais); ZUFRJ (Museu Zoologia Universidade Federal do Rio de Janeiro).

**Reference**

Bruschi DP, Busin CS, Toledo LF, Vasconcellos GA, Strussmann C, Weber LN, Lima AP, Lima JD, Recco-Pimente SM (2013) **Evaluation of the taxonomic status of populations assigned to *Phyllomedusa hypochondrialis* (Anura, Hylidae, Phyllomedusinae) based on molecular, chromosomal, and morphological approach.** BMC Gen 14:70.

Faivovich J, Haddad CFB, Garcia PCA, Frost DR, Campbell JA, Wheeler WC: **A systematics review of the frog family Hylidae, with special reference to the Hylinae, a phylogenetic analysis and taxonomic revision**. Bul. Am. Nat. Hist. 2005, 294: 1-240.

Faivovich J, Haddad CFB, Baêta D, Jungfer KH, Álvares GFRA, Brandão RA, Sheil C, Barrientos LS, Barrio-Amós CL, Cruz CAG, Wheeler WC: **The phylogenetic relationships of the charismatic poster frogs, Phyllomedusinae (Anura, Hylidae).** Cladistics 2010, 25: 1-35.

Wiens JJ, Fetzner JW, Parkinson CL, Reeder TW: **Hylid frog phylogeny and sampling strategies for speciose clades**. Systematic Biology 2005, 54:719-748.

Wiens JJ, Kuczynski CA, Hua X, Moen DC: **An expanded phylogeny of treefrogs (Hylidae) based on nuclear and mitochondrial sequence data.** Mol. Phylogen. Evol. 2010, 55: 871–882.
