# Supplementary material for: Molecular and Morphological Evidence Reveals a New Species in the Phyllomedusa hypochondrialis Group (Hylidae, Phyllomedusinae) from the Atlantic Forest of the Highlands of Southern Brazil
Source: PLoS One. 2014 Aug 20;9(8):e105608. doi: 10.1371/journal.pone.0105608 (PMC4139387; doi:10.1371/journal.pone.0105608)
Supplement: Appendix S1 — (DOCX) [file pone.0105608.s002.docx]

**Appendix S1:** List of examined specimens

*Phyllomedusa ayeaye*. BRAZIL: Minas Gerais: Poços de Caldas, Morro do Ferro (CFBH 0850); São Roque de Minas (CFBH 2953-54); Sacramento (CFBH 26621-625); Itabirito (UFMG 12476-84).

*Phyllomedusa azurea*. BRAZIL: Mato Grosso do Sul: Corumbá (CFBH 2576-2577); Corumbá, Passo do Lontra (MNRJ 17861-17879); Jardim (CFBH 3535-3542); Caarapó (UFMG 8266-8267). Goiás: São João da Aliança (UFMG 11575-11576).

*Phyllomedusa centralis*. BRAZIL: Mato Grosso: Chapada dos Guimarães (CFBH 15794-15795, 21845).

*Phyllomedusa hypocondrialis*. BRAZIL. Amapá: Amapá (CFBH 17285-17286); Pará: Belém (MNRJ 13671-13675); Canaã (CFBH 3665-3666); Juruá, Rio Xingu (MZUSP 66183, 64273-64278).

*Phyllomedusa megacephala.* BRAZIL. Minas Gerais: Jaboticatubas (UFMG 1632-1634); Santana do Riacho (CFBH 00793, 30762-30763, 30769, 30792, 30898-30899).

*Phyllomedusa nordestina*. BRAZIL: Alagoas: Arapiraca (UFMG 7579-7584). Bahia, Aureliano Leal (CFBH 18732-18734), Maracás (CFBH 19532-19534, 19536, 19538). Ceará: Crato, Araripe (MNRJ 13630, 32870). Sergipe: Campo Formoso (MZUSP 38845-38846). Minas Gerais, Buenópolis (UFMG 7423-7427).

*Phyllomedusa oreades*. BRAZIL. Goiás: Alto Paraíso de Goiás (UFMG 13840). Pirenópolis, Parque Estadual dos Pirineus (CFBH 15797-15798).

*Phyllomedusa palliata*. BRAZIL. Acre: Cruzeiro do Sul (CFBH 26208).

*Phyllomedusa rohdei*. BRAZIL. Espírito Santo: Santa Teresa (UFMG 13154-13159). Rio de Janeiro. Rio de Janeiro (CFBH 11087-11088). Minas Gerais, Catas Altas (UFMG 12582).
